# Management of Pathologic Hip Fracture Secondary to Musculoskeletal Echinococcosis: A Case Report

**DOI:** 10.7759/cureus.70195

**Published:** 2024-09-25

**Authors:** Leonidas E Mastrokostas, Paul G Mastrokostas, Mitchell K Ng

**Affiliations:** 1 Department of Orthopaedic Surgery, Maimonides Medical Center, Brooklyn, USA; 2 Department of Orthopaedic Surgery, State University of New York Downstate Health Sciences University, Brooklyn, USA

**Keywords:** drain site infection, echinococcus, hydatid cysts, postoperative care, proximal femur replacement

## Abstract

A 50-year-old female from Uzbekistan presented to our emergency department with severe right hip pain and loss of ambulation. Her history included multiple hepatic echinococcal cyst resections. After a fall, she underwent a proximal femur open reduction and internal fixation (ORIF) and revision in Uzbekistan, which revealed broken screws and cystic lesions. Subsequent treatment included hardware removal, proximal femur replacement, and antiparasitic therapy, leading to significant improvement. This case highlights the need for considering rare pathologies in atypical orthopedic presentations and demonstrates the effectiveness of integrating detailed history, careful diagnostics, and coordinated care to manage challenging conditions.

## Introduction

*Echinococcus granulosus* (*E. granulosus*) has long been endemic to Central Asia, with surgical incidence rates reaching more than 10 cases per 100,000 [1]. While infection of the liver and lungs is most prevalent, muscle involvement is extraordinarily rare, accounting for only 3-5% of cases, as lactic acid and muscle contractility create an environment that makes cyst formation improbable [2-7]. Nevertheless, intramuscular cystic echinococcosis (CE) rates are higher in those with liver or lung involvement [8]. CE can remain asymptomatic for extended periods, and bone infections are often identified only after a pathologic fracture or secondary infection, necessitating heightened diagnostic vigilance in patients with atraumatic fractures from endemic regions.

Although rare in orthopedic pathology, CE with musculoskeletal involvement has been documented. For example, a case study by Csotye et al. highlighted a locally invasive intramuscular CE presenting as a pathologic fracture in a 39-year-old man, with a large echinococcal cyst in the vastus lateralis causing erosion of the proximal metaphysis of the femur [9]. Musculoskeletal CE often requires a combination of surgical intervention and prolonged antiparasitic therapy due to the potential for cysts to invade neighboring tissues and recur if not fully eradicated [10,11].

This case report details a rare instance of a pathologic hip fracture due to musculoskeletal CE in a patient with a history of echinococcal infection. By detailing this case, we highlight the importance of considering hydatid disease in differential diagnoses for pathologic fractures. The patient was informed that data concerning the case would be submitted for publication, and she provided consent.

## Case presentation

In January 2022, a 50-year-old female from Uzbekistan presented to our ED with severe right hip pain and loss of ambulation, resorting to a wheelchair after three weeks. She reported no prior hip pain, fever, chills, weight loss, or trauma. Imaging revealed a peritrochanteric hip fracture, prompting transfer for further management. Her history included multiple hepatic echinococcal cyst resections, most recently in 2018. Despite no significant physical abnormalities, she had tenderness and pain with hip movement. Informed about the risks of an untreated hip fracture, she left against medical advice.

**Figure 1:**
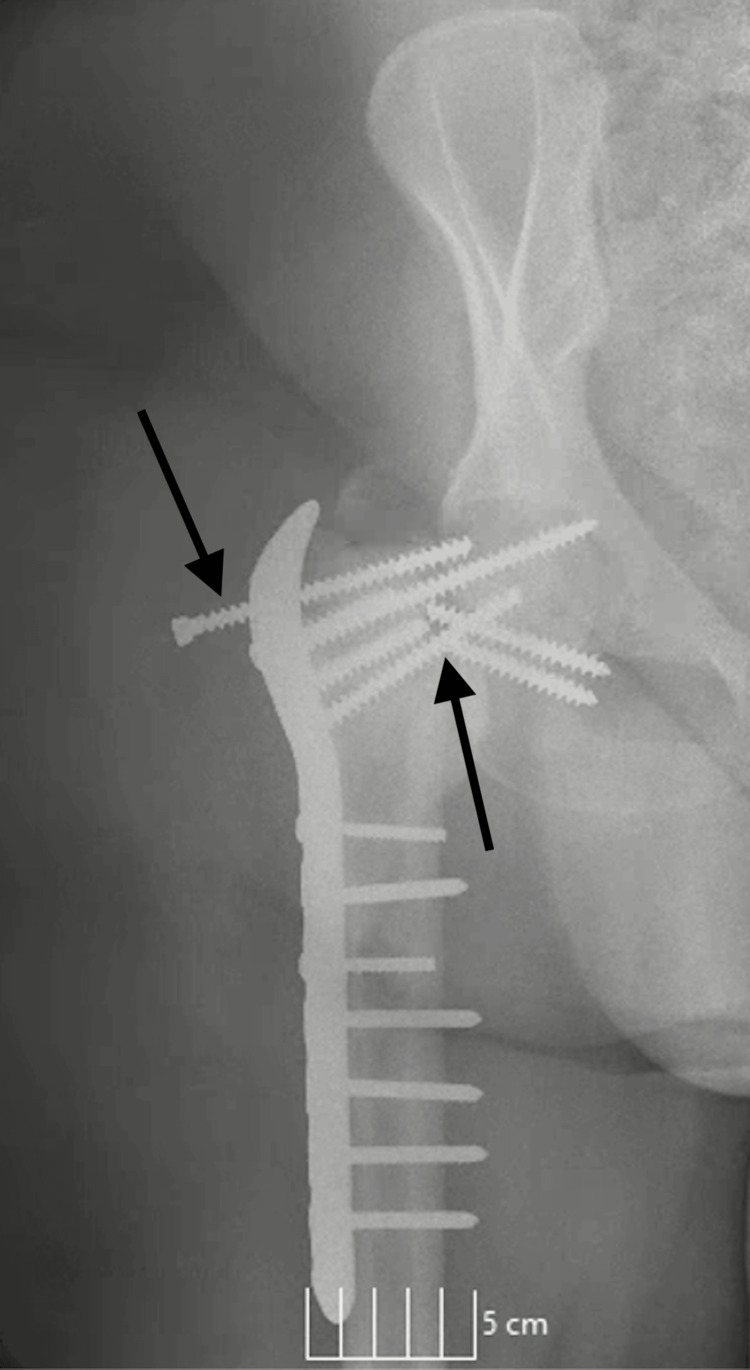
Post-ORIF revision X-ray following the fall - two broken screws traversing the femoral neck with significant retraction of one screw ORIF: open reduction and internal fixation

In September, the patient returned with aggravated right hip pain after a fall from her wheelchair. She had undergone a proximal femur open reduction and internal fixation (ORIF) in March and a revision in May in Uzbekistan, which also involved bone cyst removal. Radiographs showed several broken screws and significant screw pull-out (Figure 1).

She underwent hardware removal, placement of antibiotic beads, and a biopsy. The antibiotic beads contained vancomycin and gentamicin, which are standard choices for local infection control in cases of bone involvement. At that point, *Echinococcus* was not yet confirmed, though it was later considered due to the patient’s history of hepatic cysts.

**Figure 2:**
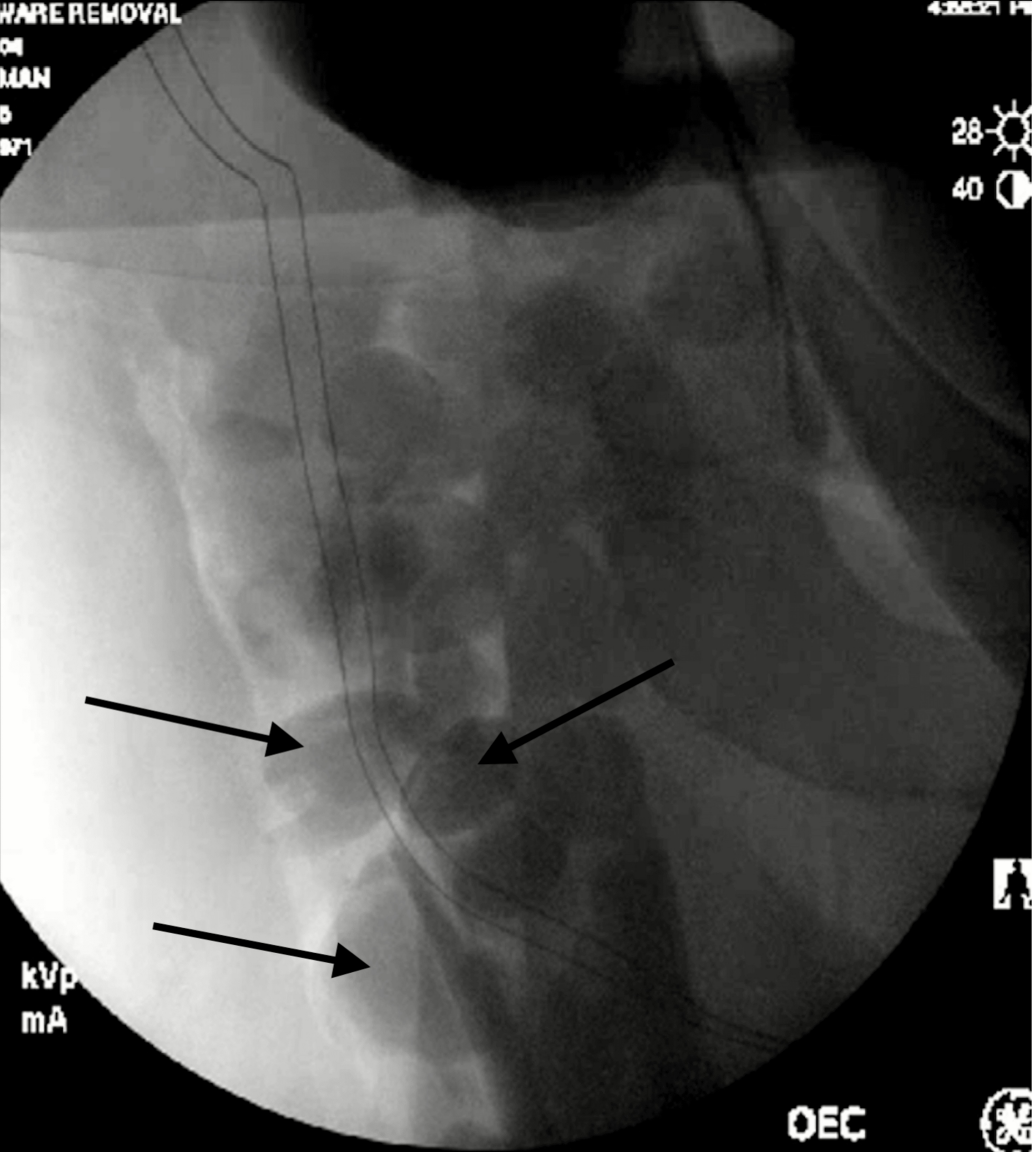
Intraoperative fluoroscopic imaging - removal of hardware and osteotomy of the proximal femoral head and neck, revealing intramuscular cystic lesions

**Figure 3:**
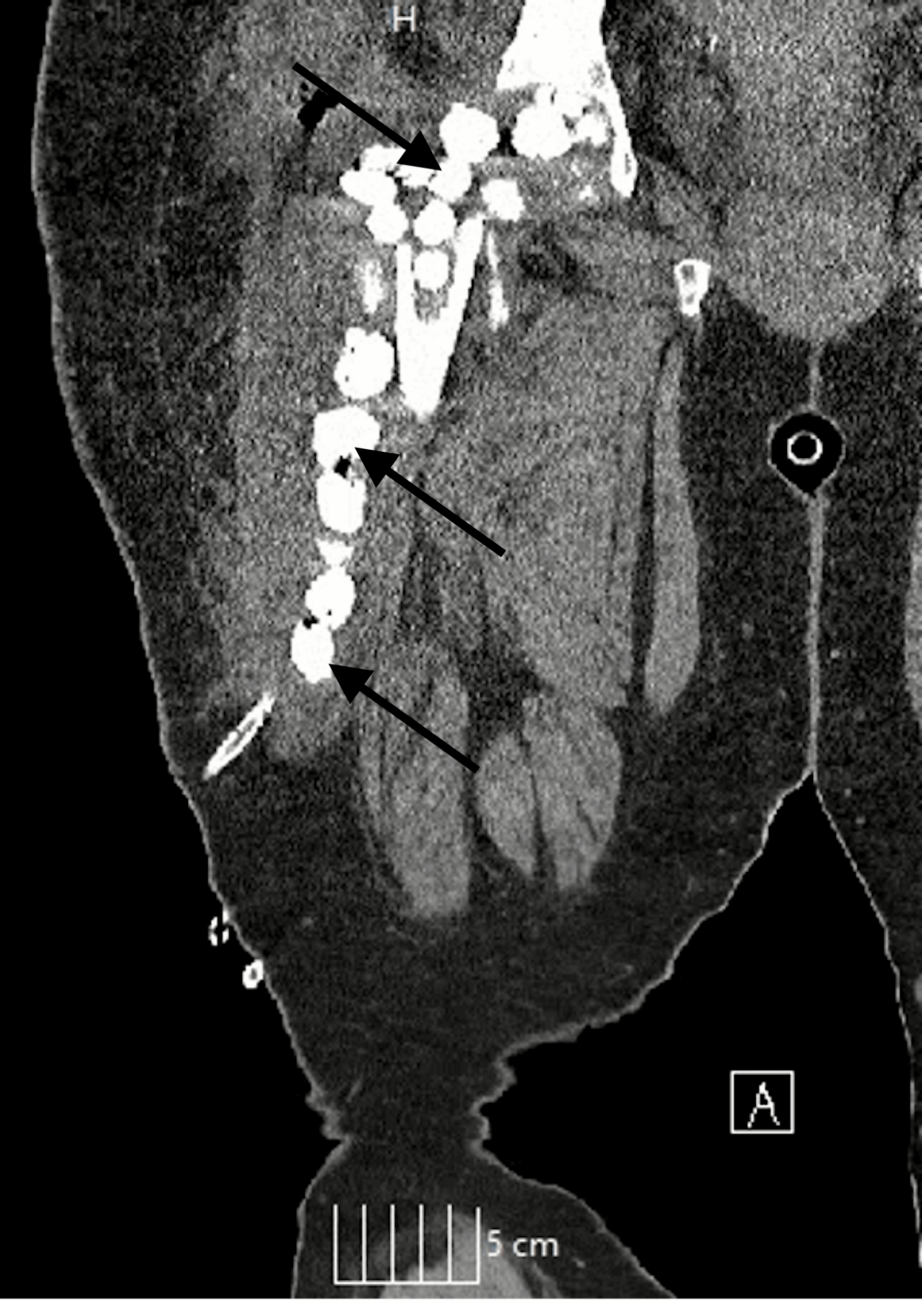
Postoperative CT scan - removal of hardware and osteotomy of the proximal femoral head and neck, displaying multiple intramuscular echinococcal cysts versus multiple abscesses

Intraoperative (Figure 2) and postoperative (Figure 3) radiographs displayed cystic lesions in the thigh.

**Figure 4:**
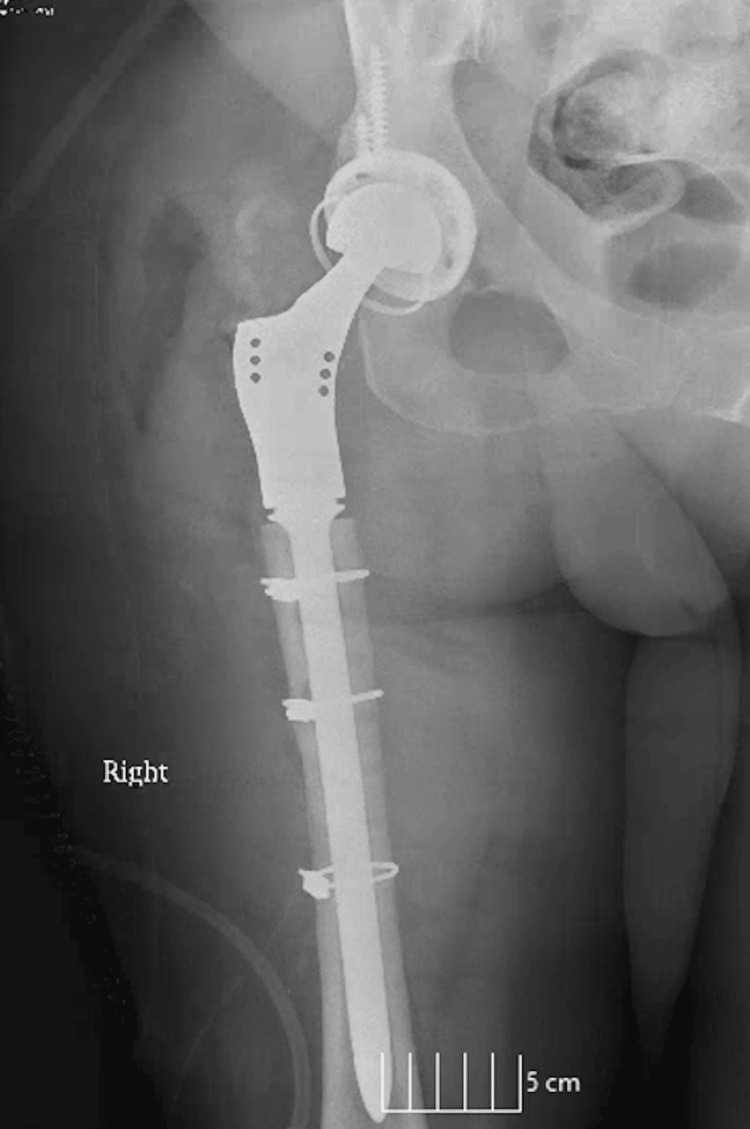
Postoperative X-ray - proximal femur replacement (PFR)

Subsequently, she had a proximal femur replacement (PFR) and tolerated the procedure well (Figure 4). A wound vac and Hemovac drain were placed, and she was started on Lovenox and vancomycin. Her postoperative check was unremarkable.

Due to suspected *E. granulosus* involvement, indicated by intramuscular cysts in the proximal right thigh on MRI, Infectious Disease was consulted for parasitic management. She was started on albendazole and praziquantel. Confirmatory Western blot testing provided definitive evidence for *E. granulosus*. Consultation with her previous surgeon in Uzbekistan revealed echinococcal involvement in the right hip during her hepatic cyst resection. Infectious disease recommended drainage and hypertonic saline injection into the cysts, continuing her anti-helminthic treatment. Interventional radiology (IR) extracted 20 cc of purulent material with a 10 French drain; cultures showed no organisms.

At two weeks postop, the lateral thigh wound was not fully healed, but the wound vac was discontinued. A one-week follow-up was scheduled for a wound check and possible staple removal. Ultimately, the patient was discharged after significant improvement and stability. At follow-up, the wound was well-healed. She reported no postoperative pain and was ambulating well with a cane, denying any fevers or chills. However, the IR drain still contained cloudy, purulent fluid. It was noted that she had not received follow-up care for the home drain. Orthopedic services contacted IR, and an appointment was made for drain removal.

Three days later, she presented to the ED with posteromedial thigh pain, a fever of 39 °C, and a foul smell from the IR drain site. Imaging revealed a large fluid collection lateral to the prosthesis. Despite these findings, the radiologist expressed low concern for communication between the medial thigh abscess and the prosthesis. The patient was started on empiric intravenous vancomycin and cefepime. Drainage procedures were performed by the IR team, along with continued albendazole and praziquantel. Interventions included evaluating drain function and aspirating fluid, revealing *Staphylococcus (S.) aureus*, moderate *Corynebacterium* species, and rare *Enterococcus (E.) faecalis* in the medial collection, but no growth at the surgical site. The antibiotic regimen was adjusted to daptomycin and unasyn, discontinuing vancomycin and cefepime. The patient was discharged home on continued anti-helminthic medication, showing significant improvement.

## Discussion

Cysts secondary to *E. granulosus* infection are often slow-growing and may not produce symptoms until they reach a significant size or begin to invade neighboring tissues, potentially causing severe health complications depending on their location [12]. These cysts tend to go unnoticed until they present in the form of pathology - the most common of which is an atraumatic hip fracture when osseous tissue is implicated [9,13]. This case report evaluated the complex interplay between recurrent echinococcal infection and orthopedic management in a patient presenting with an atraumatic hip fracture, highlighting the diagnostic challenges and the need for a multidisciplinary approach in the management of such rare presentations.

When confronted with complex pathology, obtaining a comprehensive history significantly aids clinicians in identifying underlying causes, tailoring specific diagnostic tests, and guiding treatment plans. This approach is critical in atypical presentations, as determining whether the patient has had significant medical interventions or exposure risks can dictate the treatment approach. A study by Velasco-Tirado et al. assessed recurrence rates for CE and found liver CE has high recurrence rates following surgical resection [14]. Therefore, our patient’s multiple liver cyst resections suggest a heightened predisposition for CE to manifest in less common organs such as muscle and bone. Additionally, communication with the previous treating surgeon about the presence of echinococcal cysts in the patient’s thigh underscored the essential role of comprehensive medical records in facilitating effective care. Such detailed information exchange ensures continuity of care and helps anticipate potential complications, enhancing the overall treatment strategy.

Utilizing anti-helminthic medications is crucial in managing echinococcal infections, significantly influencing both surgical and conservative treatment outcomes. In our case, the early use of albendazole and praziquantel was vital in controlling the infection and minimizing post-surgical complications. While albendazole is established for conservative treatment of CE, combining it with praziquantel is not universally recommended. However, a study by Popova et al. involving 20 patients treated with both showed improvement in 85% of cases, suggesting enhanced therapeutic effectiveness [15]. In their study, patients were treated with albendazole for three to nine months in combination with praziquantel for two to six months [15]. Yasawy et al. also found this combination more effective than albendazole alone [16]. While these findings are promising, more comprehensive studies are needed to establish the optimal regimen for CE. Alongside pharmacological treatment, hypertonic saline injections played a pivotal role. Known for its osmotic effect, hypertonic saline helps reduce the viability of echinococcal cysts, improving the pharmaceutical protocol [17]. This combined approach underscores the necessity of a tailored and multifaceted treatment strategy, integrating both chemical and physical modalities for optimal outcomes in CE cases.

Regarding the use of antiparasitics in bone cement, while antibiotics are commonly incorporated into bone cement for local infection control, the use of antiparasitics such as albendazole or praziquantel in cement is not standard practice. There is limited evidence or guidelines supporting the efficacy of antiparasitics when delivered in this manner, as they are typically administered systemically rather than being integrated into cement.

The importance of meticulous postoperative drain care cannot be overstated, especially in patients with significant risks of infection or contamination. In this case, the development of a drain site infection underscores the critical role of comprehensive postoperative care. Notably, the patient’s lack of adequate at-home drain care might have contributed significantly to this complication. This oversight highlights a crucial gap in the continuity of care from the hospital to the home setting, underscoring the need for thorough discharge planning and patient education. A study by Tschudin-Sutter et al. evaluated the risk factors associated with the development of surgical site infections (SSIs) after cardiac surgery involving sternotomy [18]. The authors found that prolonged drain retention is a significant risk factor for SSIs, highlighting the necessity of careful post-operative drain management to prevent complications.

Nevertheless, adaptability in response to emerging complications is crucial. Upon identifying the pathogenic species responsible for the patient’s newly developed abscess, a tailored antibiotic regimen was quickly employed. A systematic review by Bucataru et al. assessed risk factors leading to SSIs [19]. In the setting of an SSI, the authors detailed how postoperative wound care and monitoring are vital, with early detection, treatment, and cytology preventing more severe complications and improving outcomes. Thus, comprehensive postoperative management, including diligent drain care, thorough patient education, and adaptive response strategies, remains indispensable in safeguarding against complications and enhancing patient recovery.

## Conclusions

This case highlights the complexities in diagnosing and managing atypical echinococcal infections. Our multidisciplinary approach demonstrates the necessity of integrating advanced pharmacological treatments and meticulous surgical care. It emphasizes the importance of vigilant postoperative care and illustrates the potential for atypical presentations in patients with a history of CE. Such cases underscore the critical need for thorough patient history-taking and coordinated care across specialties to ensure the best outcomes for complex infectious diseases.
